# Factors Contributing to Leaving against Medical Advice (LAMA): A Consideration of the Patients’ Perspective

**DOI:** 10.3390/healthcare11040506

**Published:** 2023-02-09

**Authors:** Eddieson Pasay-an, Romeo Mostoles, Sandro Villareal, Reynita Saguban

**Affiliations:** College of Nursing, University of Hail, Hail 81491, Saudi Arabia

**Keywords:** leaving against medical advice, patient, qualitative, Saudi Arabia

## Abstract

It is essential to understand why patients choose to leave the hospital in direct opposition to medical advice. This understanding could help to identify individuals who are at risk of adverse outcomes. In realizing this need, this study aimed to explore the factors contributing to the decision of patients to leave the hospital against medical advice. Materials and Methods: This research employed a descriptive-analytical approach. It was conducted in the city of Hail, Kingdom of Saudi Arabia. The participants were 13 patients who had opted to leave against medical advice from the Emergency Department of the government-subsidized hospitals. The researchers employed both purposive and snowball sampling. In snowball sampling, the researchers used referrals from initial participants to generate additional participants. Moreover, purposive sampling was used to find the individual that would best contribute to addressing the research issue. The data gathering was conducted from April to June 2022. Results: Five themes emerged based on the accounts of the 13 participant patients. These included (1) health literacy, (2) self-diagnosing, (3) unclear explanations regarding their condition, (4) prolonged waiting times, and (5) communication issues. Conclusion: The factors contributing to patients leaving against medical advice resulted in the five themes mentioned above. While interactions between patients and healthcare professionals may be challenging, pertinent health information must still be handed down clearly to patients.

## 1. Introduction

Leaving against medical advice (LAMA) remains a major source of low-quality healthcare, accounting for up to 2% of all hospital discharges [1]. In the emergency room, which is the busiest facet of hospitals, 1–3% of LAMA visits are due to incomplete emergency care, walkouts, and departing against medical advice [2]. Because it subjects them to legal and ethical ramifications, managing this issue is particularly challenging [3] for healthcare team members. Moreover, LAMA reduces the capacity of the patients both to make such a choice and to grasp its potential repercussions. In such cases, there is a need to improve the patient’s knowledge of the risks and to establish and strengthen their understanding of the effects of leaving the hospital, including, for example, effective communication between patients, doctors, and other medical professionals [4], and informing hospitalized patients and families of the negative effects of LAMA [5].

Due to its negative effects, no hospital actually permits LAMA; nonetheless, it has emerged as among the most prevalent issues in the present healthcare system [6]. In general, patients who leave against medical advice on their discharge have a higher risk of hospital readmission, even mortality [7], and longer hospital stays after readmission [8]. Therefore, to promote compliance and avoid the negative effects of defying medical advice and leaving the hospital, it is advantageous for the medical personnel to establish a strong rapport with patients who are more likely to LAMA [9]. Along with provider- and environment-related stress, patients who opt to LAMA may additionally encounter a number of personal, societal, and financial obligations [10]. Despite being poorly explained, communication, medical, and psychosocial problems are important factors [7]. In earlier investigations, the reasons charted for LAMA only included such things as financial concerns, waiting time, lack of medical progress, and wanting medications [5,11,12]. These reasons were evaluated retrospectively and quantitatively. To the researchers’ knowledge, few studies have been done on LAMA, and none have looked at the patient’s own experiences of the factors influencing their decision to discontinue therapy. Therefore, it is crucial that this study be done in order to further our understanding of LAMA.

It is essential to understand why patients choose to leave the hospital when doing so is in direct opposition to medical advice. This understanding could identify individuals who are at risk of adverse outcomes if they LAMA, thereby enabling earlier intervention to lower excess morbidity, mortality, and medical costs, thus having a significant positive impact on both the patient’s quality of life and the financial health of the healthcare systems. With these considerations, this study aimed to answer the research question of what factors lead to patients making the decision to leave against medical advice.

## 2. Materials and Methods

### 2.1. Study Design

This research employed a descriptive-analytical approach to explore the factors contributing to patients leaving against medical advice. The idea that the analysis of themes is intended to look for common or shared meanings served as the basis for its application. According to Braun and Clarke [13], thematic analysis is an acceptable and efficient method to use when attempting to understand a group of events, concepts, or deeds within a data set.

### 2.2. Setting

This study was conducted in the city of Hail, Kingdom of Saudi Arabia. The participants included 13 patients who had opted to leave against medical advice from the main government-subsidized hospitals in the city of Hail. The researchers used purposive and snowball sampling. In snowball sampling, the researchers used referrals from initial participants to generate additional subjects. For example, the researchers asked participant 1 if she/he knew someone who opted for LAMA. This process was used until the researchers reached saturation point. The inclusion criteria were patients who (a) left the Emergency Department but who were advised to stay for confinement from January to December 2021, (b) were in good health, (c) were able to speak and comprehend English, and (d) were willing to participate. Table 1 presents the demographics of the 13 participants.

The researchers were guided with semi-structured questions that were piloted to check the validity of the questions. In the face validity, the researchers invited three panels who are experts in qualitative research. The researchers assessed if the instrument looked appropriate and had pertinent items on it. The interview began with an open-ended inquiry to foster trust and enhance communication between the researchers and the participants. The participants were asked for permission to record the interviews before the one-on-one interview commenced. The grand question was, “Can you tell us about your experience in leaving the hospital against medical advice?” The researchers used probing questions to clarify and explore the answers of the participants further. The participants were also asked to provide pertinent examples from their own experiences to help explain the concept behind their responses. Each interview lasted between 40 and 50 min.

### 2.3. Data Collection

The participants were initially sent a letter of invitation so they could decide whether to volunteer to participate or not. They were given a consent document to sign before participating. The participants determined the time, place, and date of the interview. One female and three male researchers conducted the interviews. All of the interviewers had extensive expertise in conducting interviews and were employed in academia at the time of the investigation. The researchers developed an empirical conviction that a category was saturated when they repeatedly observed instances that were alike and no new data was being discovered. Data saturation was reached on the 13th participant in this study which came out from recollecting similarities in the interviews. The data gathering was conducted from April to June 2022.

Two researchers worked independently to analyze the interview materials and summarize, extract, and develop the themes that emerged. The study team discussed and settled divergent views on the content of the themes. The researchers devised the protocols and procedures for the study. They confirmed the quantity of the data that they had gathered, and the participants verified it to ensure the validity of the findings. This was done to ensure that every detail of their descriptions of their unique situations was recorded. Lincoln and Guba [14] suggested four criteria for developing the trustworthiness of a qualitative inquiry: credibility, dependability, confirmability, and transferability. The researchers followed these criteria throughout the investigation.

### 2.4. Ethical Consideration

This study had approval and ethical clearance from the Ethical Review Board of the Ministry of Health (IRB-2021-32). The confidentiality of the data was guaranteed by the researchers. The personal information of the patients was kept completely confidential.

### 2.5. Measurement

Thematic analysis, a technique for assessing qualitative data, was used to examine the data gathered for this study. This approach is used to find, examine, and understand the internal hidden patterns and themes in qualitative data. The researchers followed the steps in the process of theme analysis provided by Braun and Clarke [13]. The steps were as follows: (1) familiarizing oneself with the complete data set, which requires frequent and active reading of the data, (2) making notes on potential data items of interest, queries, linkages between data items, and other early concepts, (3) examining the coded and compiled data extracts to determine whether there are any possible themes that have a bigger impact, (4) conducting a two-level analytical method in which the researcher examines coded data positioned within each topic at the first level of analysis to guarantee a proper fit, and (5) improving the thematic map and defining and describing each theme narratively, explaining why it is significant to the larger research question. Then the final report’s theme titles were examined to ensure that they were succinct and appropriately descriptive. The final step (6) was creating the manuscript or report and writing up the final analysis or summary of the findings [13].

## 3. Results

Five themes emerged based on the accounts of the 13 participant patients. They included (1) health literacy, (2) self-diagnosing, (3) unclear explanations for their condition, (4) prolonged waiting times, and (5) communication issues.

### 3.1. Health Literacy

Health literacy emerged as a theme that pertains to the capacity of the patients to acquire and comprehend the instruction or education given to them. Patients who go to the Emergency Department and are advised to be admitted may find it challenging to understand basic health information, such as the reason for the confinement and health intervention. Such concepts were clearly articulated by the patients during the interview, as seen in the following examples.

P9: Too much effort to wait in that area (Emergency Department), but at the end, they say that I will be staying for a night for observation and I did not get the reason for my admission.

P6: I went home despite the doctor’s advice that I have to be admitted because I just thought that I would be okay. It turns out that I did not grasp the doctor’s explanation regarding my condition… Anyway, it was a lesson to learn.

P7: While the nurse was explaining why she puts wires in my chest… I got uncomfortable as I felt that I might be electrocuted… so shameful that I left the ED just because of that experience. I went home, but after three days, I went to another hospital and was referred to the outpatient department, and the same procedure was done.

### 3.2. Self-Diagnosing

The patients’ own impressions of their medical problems, which they frequently misunderstand, are related to self-diagnosing. Some patients may make a diagnosis for themselves using data that they have gathered from the internet, their own experiences, or their capacity to identify the warning signs or symptoms of a disease that a family member has already experienced. Understanding one’s own sickness is a critical cognitive aspect that has been repeatedly stressed. Moreover, it can have a substantial impact on how effectively a patient adapts to their condition, how the disease evolves, and how it is treated. The patients mentioned the following in their accounts.

Px 1: I just visited the Emergency Room to get medicine because I felt my heart beating fast and it seems I felt so tired. The doctor instructed me to stay, but I thought that was an over fatigue, like what my friend was experiencing… If you were in my shoes, you would decide to go home if you felt okay, right?

Px 4: I Googled my symptoms before I went to the hospital because I just really only wanted my maintenance medication to be dispensed, but the doctor referred me to the ED and the ED doctor told me to stay for observation. I told the doctor that I would just go back.

Px 6: You know, I had to leave the hospital even though the doctor told me to stay. One of my children had the same symptoms as me, and the doctor let him go. The attending nurse explained to me as well, but I really wanted to go home. I insisted, so they just let me sign the waiver.

### 3.3. Unclear Explanation of the Condition

The unclear explanation of the patient’s condition pertains to a situation in which the patient does not comprehend the explanation given by the healthcare providers. In context, it prompts the patients to leave the hospital because they have misinterpreted the explanation of their chief complaint. In LAMA cases, it is crucial to know whether the patients can understand their diagnosis and treatment plan. At times, medical personnel overestimate how well the patients comprehend their explanations.

Px 12: The only complaints I had were rashes and I felt pressure when I was breathing. The nurse said that I was okay because it was just an allergy and I needed to be observed for a while. However, the doctor said that I would be injected with something… I didn’t know that medicine… I began to question it because the nurse had said that it was just an allergy… That is why I got mad and transferred to another hospital.

Px 13: The doctor on duty explained that my situation could be asthma and the other one said that it was a respiratory disorder. I got confused about which was the correct one for my condition, or did I get it right? I don’t know.

Px 3: In my case, I did not really understand the doctor’s explanation regarding my disease because every word was new to me… I requested not to be admitted. When I got to the primary health center with the help of my friend, that doctor’s explanation was the same as the one of the doctor in the hospital. However, it was my mistake that I reacted right away.

### 3.4. The Waiting Time

The waiting time refers to the time that the patients have to wait to be attended to by the medical personnel after the information about their condition has been relayed by the interviewer. In most cases, the long wait times have a negative impact on the patient’s willingness to go back and are significant contributors to their dissatisfaction. In the interview, most of the patients expressed dissatisfaction regarding the time that they were in the waiting area. Example comments include the following.

Px 8: I understand that medical officers and nurses have so many patients but I think they forgot to attend to me. The only thing that they were doing to me during that time was looking into the monitor and asking if I was okay.

Px 11: I went home because I cannot wait that long to be attended. Although, the doctor said that I would be admitted for observation, it seems that the process is too long and I do not want to keep waiting… I hate that kind of situation.

Px 2: I asked my brother to fetch me in the Emergency Department because I could no longer wait for the attendant to visit me in the observation room… Although the nurse assured me that my blood pressure had already decreased because of the medication, but that should not be the reason for me to stay and wait for the final discharge.

### 3.5. Communication Breakdown

This theme relates to the language barrier that most of the patients considered as enabling them to leave the hospital. The communication breakdown includes how the message was relayed to them and how the patient understood the message. Linguistic differences between healthcare professionals and patients could be a deciding factor for the patients not being satisfied with the care, thereby searching for a better hospital to help them. The following comments are representative of patients’ claims.

Px 10: The nurse did not understand what I was saying. She always asked her friend to interpret what I was saying. I felt offended… I cannot help comparing the hospital to other hospitals where I have been before…

Px 5: While I was waiting my turn to be attended, a woman in white kept mentioning words, something that I didn’t understand. She seemed to be talking fast, which I could not grasp at all. I asked her to slow down a bit, but I think it was her practice. I asked another to attend to me, but they were the same… My sister suggested transferring to another hospital.

Px 7: It was my first time to stay so long in the Emergency Department for observation purposes, but I was not really comfortable whether my nurse understood me or whether I understood what she was saying. I think we had problems with communication, so I requested to have another nurse.

Px 9: I left the hospital just like that because I felt like the next time when I explain I will have to use sign language. I think the attendant is very new and she cannot understand…

Px 10: At first, I could not express myself with the language they were using. I asked my friend if she could help me transfer to another hospital.

## 4. Discussion

Leaving against medical advice remains a problem that has a negative impact on patient outcomes, the economy, and hospital resources. It affects a wide range of patients. Despite the fact that this issue has a variety of root causes, some tactics have been found to effectively reduce the number of patients who defy their doctors’ orders and leave. This study aimed to explore the factors contributing to patients who LAMA. In this present study, four themes emerged: (1) self-knowledge, (2) unclear explanations of the condition, (3) waiting time, and (4) communication issues.

The emergence of health literacy as a theme in this study suggests that, despite patients being given the information, they appeared not to follow instructions and were thereby unable to make decisions regarding their health. This influences how these patients live their lives and how they access, use, and keep up with their healthcare routines. According to Peerson and Saunders [15], health literacy is regarded as both a hindrance and a facilitator, an influencer, a byproduct, and a result of, among other things, excellent health, education, social policy, and programs. A person’s overall welfare is promoted through health literacy. An individual’s level of health literacy influences their decision-making and their actions in their family, workplace, and their community. Jessup and colleagues [16] maintain that patients must have a sufficient level of health literacy to interact and communicate with their healthcare providers, manage their medications and appointments, actively monitor their conditions, adjust regimens in response to changes in the course of their diseases, and implement and sustain lifestyle changes for the management and improvement of their health. On the basis that the patients were provided with the information but did not follow the recommendations and had difficulty making decisions about their health, this study finding suggests that healthcare workers must ensure clear information and activate effective communication.

In this study, self-diagnosing was one of the contributing elements to LAMA. This indicates that the patient’s knowledge of their disease was in some way based on their own comprehension, which caused them to lack understanding of their therapy, resulting in conflict with the healthcare professional. In effect, treatment options may be limited by one’s insufficient or incorrect understanding of their illness [17]. Accordingly, the accuracy of this knowledge can vary when compared to the current state of objective medical knowledge. Indeed, in the common opinion, patients who are both more informed about and have a better comprehension of their condition adhere to their therapy more closely [18]. This indicates that healthcare professionals need to discern the understanding of the patient before they make their decision. Therefore, the need to improve patient knowledge is of importance that is assumed to improve their compliance with medical advice tremendously.

Unclear explanations of the patient’s condition were another factor that contributed to LAMA. Here, patients do not have the necessary information regarding the side effects and outcomes of medical decisions. Unclear explanations could be brought about by improper word choice, possibly because of presumptions made regarding the patient’s degree of health comprehension, gain resulting in LAMA. A patient’s decision to LAMA has a range of negative repercussions, including increased medical costs, readmission, and complications for the patient and significant others [10]. This study’s finding contributes to the information and decision-making of the medical staff in that they must make the individual patients more aware of any potential consequences of their choices. It is crucial to instill a culture of compliance with the recommended therapeutic procedure among the populace, to improve the welfare amenities of hospitals, to monitor the performance of the medical staff, and to strengthen the bond between the patient and the medical team to eliminate or reduce the likelihood of the occurrence of the problem of LAMA. Increasing knowledge and actively engaging in communication that is aimed at improving patient-physician interactions and shared decision-making regarding patient care is a technique for lowering the prevalence of premature discharges.

Waiting time has also been claimed to be a factor in leaving against medical advice. This suggests that patients’ care was judged to have been of lower quality or standards in terms of waiting times. LAMA is frequently influenced by the length of time waiting for the next steps in the admission process. This result is in accordance with those of earlier studies [19,20,21]. Likewise, Sharif et al. [22] found that patients complained of long wait times, not having their physical health assessed, and prejudice. Ibrahim et al. [1] additionally revealed that some patients leave the hospital against medical advice because of the lengthy administrative processes involved in consultation rooms and discharge procedures.

The communication issue has also emerged as a theme to be a factor in leaving against medical advice. This indicates that a patient’s decision to LAMA signifies a communication breakdown that needs to be resolved. This present study concurs with the study of Onukwugha et al. [10], which maintained that the patients felt that they spent a long time waiting in the hospital. It is noteworthy that the communication issue and LAMA decreased significantly by improving patient-centered quality of care, physician-patient communication, patient engagement in treatment, and patient satisfaction [23].

Overall, the findings of this study can help healthcare professionals better understand why patients disregard medical advice. Tailor fitting these identified factors in this present study results with health education and planning can also be used to establish the appropriate treatments to prevent early discharge.

### Study Limitations

This study has its limitations. The 13 individuals did not adequately reflect the majority of the total LAMA patients. Hence, the findings cannot be generalized. This can be addressed by conducting a further study considering the mixed method. Moreover, the researchers have not explored the diagnoses of the 13 patients, their educational attainment, and financial status. Such variables can provide more robust information on leaving against medical advice. A further study could address these limitations.

## 5. Conclusions

Factors contributing to leaving against medical advice resulted in five themes: (1) health literacy, (2) self-diagnosis, (3) unclear explanations of their condition, (4) prolonged waiting times, and (5) communication issues. While the interactions between patients and healthcare professionals may be challenging, the health information must be handed down clearly to the patients. Limitations of this study can be carried out into another interrogation by future researchers. Moreover, this research recommends health authorities tailor-fit a program addressing the factors that contribute to leaving against medical advice.

## Figures and Tables

**Table 1:** Demographic particulars of the participants.

Demographic Information	Age	Sex	Nationality	Marital Status
Px 1	43	Male	Indian	Married
Px 2	38	Female	Egyptian	Married
Px 3	44	Female	Saudi	Married
Px 4	39	Male	Saudi	Married
Px 5	50	Female	Filipino	Married
Px 6	35	Female	Filipino	Single
Px 7	37	Male	Indian	Married
Px 8	30	Female	Filipino	Single
Px 9	38	Male	Saudi	Married
Px 10	40	Female	Filipino	Married
Px 11	35	Male	Pakistani	Single
Px 12	36	Male	Saudi	Married
Px 13	44	Female	Saudi	Married

## Data Availability

Not applicable.
